# Reduced chick performance makes supernormal clutches maladaptive in a shorebird

**DOI:** 10.1038/s41598-026-37872-6

**Published:** 2026-02-04

**Authors:** Oddvar Heggøy, Kees Wanders, Terje Lislevand

**Affiliations:** 1 Department of Natural History, University Museum of Bergen, University of Bergen, Bergen, Norway; 2 Natural History Museum of Denmark, University of Copenhagen, Copenhagen, Denmark; 3 Department of Life Sciences, Milner Centre for Evolution, University of Bath, Bath, UK

**Keywords:** Incubation limitation hypothesis, Clutch size evolution, Precocial birds, Shorebirds, Postnatal effects, Ecology, Ecology, Evolution, Zoology

## Abstract

Life-history theory predicts a trade-off between the number and quality of offspring, and that clutch sizes may be adjusted in relation to environmental conditions. In many bird species, however, clutch size is remarkably consistent, in which case factors constraining clutch size evolution are more debated. A classic example is found in shorebirds (Charadriiformes), where clutch size is typically fixed at four eggs across species and environments. Here, we experimentally enlarged clutches of Common Ringed Plovers *Charadrius hiaticula* to test the hypothesis that clutch sizes are constrained by parental incubation capacity. While most previous tests of this idea compared hatching success between clutch sizes, often with mixed results, we also investigated potential post-hatching effects. We found that chicks from enlarged clutches were consistently smaller during the first two weeks of their lives and showed higher mortality rates compared to control chicks. Although hatching success was relatively unaffected, enlarged clutches experienced reduced mass loss indicating slower embryonic growth, required longer incubation periods and hatched more asynchronously than controls. These findings suggest that laying an extra egg would be a waste of resources, with negative effects outweighing potential benefits. Our results highlight the potential importance of previously overlooked post-hatching constraints in shaping clutch size evolution in shorebirds.

**Supplementary Information:**

The online version contains supplementary material available at 10.1038/s41598-026-37872-6.

## Introduction

Studies of avian clutch size have been highly influential in the development of evolutionary life history theory and our general understanding of the variation in fecundity among organisms^1,2^. In many birds, clutch size is considered one of the most important regulators of reproductive success and lifetime fitness^1,3^, representing a balance between the benefits of producing more offspring and the costs associated with parental investment, resource availability and environmental conditions^4,5^. Many birds show considerable intraspecific variation in clutch size, related to both intrinsic (e.g., individual quality and longevity) and extrinsic factors (e.g., environmental conditions, food availability, and predation risk)^6–8^. However, in some precocial species such as most shorebirds, clutch size is truncated at the modal number of eggs, even though females are fully capable of producing more^1,9^. Despite extensive scientific interest, the underlying selective forces constraining clutch size in precocial birds are still debated^2,10,11^.

According to the *Incubation Limitation Hypothesis* (hereafter ILH)^1,10,11^, clutch size may be limited by parental capacity of efficient incubation. To ensure normal growth and development of the embryo, bird eggs must be kept within an optimal range of temperatures (usually 36–39 °C) throughout the incubation period, usually achieved through contact incubation where heat is transferred from a parent bird’s abdominal brood patch to the eggs^12^. However, if clutch size is limited by parental incubation capacity, any clutch size larger than the optimal number of eggs should be expected to result in suboptimal incubation temperatures^1,10^. Even small deviations (< 1.5 °C) from the optimal incubation temperature could have negative fitness consequences. First, both higher and lower incubation temperatures might affect growth, development, and survival of the embryo^13^. Reduced incubation temperatures may slow down embryonic metabolism and reduce embryonic growth rate^14^, which reduces the production of metabolic water and may reduce mass loss of eggs during incubation^15,16^. Reduced growth and development may also result in prolonged incubation periods^17,18^, which in turn increases the time that eggs are exposed to predators, potentially leading to elevated predation rates^1,10,19^. Second, reductions in incubation temperature may cause a decline in body condition and structural size, as well as an altered body composition, of newly hatched chicks^20,21^. Third, suboptimal incubation temperatures could have various effects that carry over to affect neonate phenotypes after hatching^11,19,22^, including reduced rates of postnatal growth and survival^13,21,23,24^. Effects might be severe for chick performance, especially in precocial species where parent birds are unable to compensate for a bad start by increasing feeding rates of young.

Shorebirds (Charadriiformes) have traditionally attracted much attention by ecologists who study clutch size evolution. Most shorebirds lay a maximum of four eggs per clutch and have precocial young^9,25^. Several experiments have been carried out to test predictions of the ILH by studying incubation and hatching success after adding an extra egg to shorebird clutches^10,26–28^. These studies have produced mixed results, typically showing prolonged incubation periods but less consistent effects on hatching synchrony and hatching success. However, Arnold^10^ showed that the combined effects of slower embryonic development, higher rates of hatching failure, and elevated egg predation risk could make larger-than-normal clutches maladaptive. Another possibility is that clutch enlargement could have post-hatching effects in shorebird chicks, although this possibility has received much less attention in the literature. In support of this idea, Larsen, et al.^26^ found that experimental clutch enlargement reduced hatchling body condition in Northern Lapwings *Vanellus vanellus*. This was expected if the extra egg resulted in reduced incubation temperatures and a higher total energy expenditure of the embryo^20,29,30^. However, nothing was known about the performance of chicks after they left the nest. As far as we know, only Lengyel, et al.^27^ has studied this aspect in shorebird chicks. In their cross-fostering study of Pied Avocets *Recurvirostra avosetta*, chicks reared by parents that incubated an enlarged clutch survived less well than those reared by control parents. The authors suggested that clutch enlargement had resulted in increased costs of incubation for the parents, reducing their ability to compete for areas which provided optimal foraging opportunities for the chicks.

We tested predictions of the ILH by experimentally manipulating clutch size in a widespread Eurasian shorebird, the Common Ringed Plover *Charadrius hiaticula* (hereafter Ringed Plover). If parents are not capable of efficiently incubating an enlarged clutch, we predicted prolonged incubation periods, more asynchronous hatching, and increased hatching failure rates in enlarged five-egg clutches compared with natural controls of four eggs. We also expected to find reduced mass loss rates in eggs from enlarged clutches because of slower embryonic development^20,30^. Importantly, and without manipulating brood sizes, we further compared the performance of young from the two experimental groups. We predicted that young from enlarged clutches should be structurally smaller and show relatively low body condition at hatching, and reduced rates of growth and survival after leaving the nest. By this approach, we aimed to clarify whether costs of enlarged clutches extend beyond incubation to offspring growth and survival, highlighting how carry-over effects between incubation and chick stages shape clutch size evolution in shorebirds.

## Materials and methods

### Field procedures and model species

Field work was performed in a study area (c. 35 km^2^) situated along the east coast of the Varanger Peninsula, NE Norway (70.25 N, 30.60 E) 28 May–15 July 2022. Most first clutches of Ringed Plovers are here completed in early June (median 2022–2024: 5 June, *n* = 138). The typical clutch size of four eggs (2022–2024: mean: 3.9 ± 0.36 (SD) eggs, range 2–4 eggs, *n* = 157) is usually completed in 6.5 days and incubation lasts for about 24 days from laying of the ultimate or penultimate egg^31^. Both sexes care for the clutch and brood^32,33^.

Nests were located from the shore and up to 2.2 km inland and 125 m a.s.l., mostly in habitats dominated by barren open ground and/or sparse vegetation (quarry: *n* = 15; roadside: *n* = 13; tundra: *n* = 9; sand dunes/beaches: *n* = 9). We used sliding calipers to measure egg size (length, width; to the nearest 0.1 mm), and a high-precision electronic scale (Tanita 1210, TANITA Corporation) to measure egg weight (to the nearest 0.001 g). The scale was placed in a plastic box covered by a lid to prevent wind disturbance during weighing. Egg weights were taken whenever a nest was found and re-measured once or twice to compare weight loss between experimental groups^16^. The timepoint of weighing was not standardized, but there was no difference between experimental groups in the time of the first weighing (Mann-Whitney *U* test, *p* = 0.23). When nests were found after clutch completion, we floated eggs to estimate hatching (and laying) date following the procedure of Liebezeit, et al.^34^. Estimated laying dates, defined as the date when the last egg of a clutch was laid, ranged from 24 May to 15 June (4 June ± 4.5 days, *n* = 46). We placed temperature loggers (Tinytag Talk 2 with a thermistor probe PB-5005, Gemini Data Loggers, or MSR 145, MSR Electronics GmbH) in all nests to record hatching events. Probes were placed in the bottom of the nest scrape and positioned in the center of the clutch. Hatching events were typically seen as sudden drops or increased fluctuations in temperature. This was often accompanied by a brief rise in humidity in nests with humidity sensors, probably due to moisture released during hatching. The reliability of this method was confirmed by visual observation of hatching in some of the nests. As we did not standardize the positioning of thermistors in the nest cup, most likely resulting in a large degree of noise in the incubation temperatures, we do not compare this variable between experimental groups. All experimental nests originally contained four eggs. We paired these nests based on estimated laying dates before randomly choosing one of them to be experimentally enlarged by one egg and keeping the other as a control (*n* = 24 pairs, but excluding two control nests where a forth egg was never laid; i.e., 46 nests were included in total). Clutch enlargement was done by using a fake egg made of FIMO modelling clay (STAEDTLER, Germany), which has similar thermal properties as real bird eggs^35^. Clutches were enlarged between 0 and 11 days from the estimated onset of incubation (week 1: 17 clutches, week 2: 7 clutches; mean ± SD: 4.69 ± 3.83 days). Clutch enlargement was performed at the same time as we placed the temperature logger, ensuring that control and enlarged clutches received the same amount of disturbance. The frequency distribution of nesting habitats (see above) did not differ between experimental groups (Fisher’s exact test, *p* = 0.36), and nests in both groups were placed at similar distances to the sea (median (IQR) control: 292 m (133–554 m), enlarged: 219 m (215–1039 m)).

We trapped incubating birds using a self-triggered spring trap. Adults were sexed from plumage characteristics^36^ and marked with a metal ring (right tarsus), a red colour ring (right tibia) and a yellow flag engraved with one letter and two numbers (left tibia) to facilitate field identification. Both partners in a pair remained together caring for all chicks most of the time, and broods could be readily identified because at least one parent was marked with a flag in all families.

Approaching the estimated hatching date, we checked nests daily to visually record hatching events. Hatching date was defined as the day when the first egg in a clutch hatched. We calculated the incubation period as the time between the estimated laying date (based on egg floatation or egg laying records) and the observed hatching date. Hatching asynchrony was measured as the time between the first and the last chick emerged from the eggshell. This was primarily estimated based on temperature logger data, supported by brief nest visits 2–3 times a day when approaching the estimated hatching date. We counted unhatched eggs as “failed” if they were left cold in the nest > 3 days after the last hatching event. Failed eggs were collected, and their contents inspected to determine approximate embryo age at the time of failure. We measured body mass of birds (accuracy 0.001 g in chicks, 0.5 g in adults) and used calipers to measure tarsus length, bill length (down/feathers to tip) and total head length (to the nearest 0.05 mm)^37^. During events of harsh weather conditions, some of the chick measurements were skipped to limit handling time. We collected blood samples from the tarsal vein of chicks and stored this in 100% ethanol until DNA extraction via ammonium acetate precipitation^38^. We performed molecular sexing by PCR amplification of the Chromo-Helicase-DNA binding protein gene (CHD), using 2602 F and 2669R primers^39^. Newly hatched chicks were too small to mark with metal rings, and we used permanent marker pens (green, blue, red, black) to colour their legs for individual recognition upon recapture. Chicks were marked with metal rings after reaching an age of approximately 3–4 days.

Every 2–4 days we tracked down broods by searching the surroundings of the nest for parents giving alarm calls. If one or more chicks from a brood were not observed with their parents after two subsequent and thorough searches, we concluded that they were dead. Before recapture, we observed broods from a distance to count surviving chicks and register their approximate positions. We used thermal binoculars (Pulsar Merger LRF XP50, Yukon Group) to locate the chicks if needed. Recaptured chicks were identified (based on leg colour and metal ring ID) and re-measured, and notes were taken on the habitat where the chicks were found. For simplicity, habitat was categorized as “terrestrial” (inland, tundra and mountain habitats) or “marine” (beach and shoreline habitats).

### Ethics declaration

Permission to carry out the experimental protocol on live animals, including the collection of blood samples, was given by the Norwegian Food Safety Authority (FOTS ID 26547). Catching and marking of birds was performed under the permission of the Norwegian Ringing Centre (license no. 1104). All methods were carried out in accordance with relevant guidelines and regulations and are reported in accordance with ARRIVE guidelines (https://arriveguidelines.org).

### Permission statement

The experimental procedure mostly took place on Governmental land and did not require any special permits from local landowners. However, we informed the Regional Nature Surveillance and local police about our activities. The experimental protocol was approved by the Norwegian Environment Agency (ref. 2022/5299).

### Data analyses

We performed statistical tests in R version 4.1.1^40^. All statistical tests were two-tailed with α = 0.05. Summary statistics are reported as means ± SD if not otherwise stated. Model assumptions were checked visually.

In a preliminary analysis we added time of clutch enlargement (days from clutch completion) as a predictor in regression models including enlarged clutches, and sex of chicks to check for significant effects on our response variables. As we found no evidence of such effects, we did not consider these variables further.

Precocial birds in general show a slight increase in the rate of egg mass loss throughout the incubation period, which is closely linked to the rate of embryonic development^15,16^. This was confirmed also in our sample, as our data was better fitted with a quadratic rather than a linear curve. To check for differences in mass loss between experimental groups, we fitted a linear mixed effect model (using the “lmer” function in the “lm4” package^41^ with egg mass as response, experimental group, linear and quadratic terms of days from the onset of incubation, and laying date as fixed effects and egg ID and brood ID as random effects. We calculated egg volume, *V*, based on the formula of Väisänen, et al.^42^:

$$V = {\text{ }}0.{\mathrm{47}}0{\text{48 }}^{*} LB^{{\mathrm{2}}} {-}{\text{ }}0.{\mathrm{269}}$$

Where *L* is egg length and *B* is egg breadth. To compare the rate of egg mass loss between experimental groups we added an interaction between days from the onset of incubation and experimental group to the model. We excluded data collected from clutches > 22 days old for this analysis, as only experimentally enlarged clutches were represented.

We used the “Nest” model in the R package “RMark”^43^ to calculate Daily Survival Rates (DSR) of nests and checked for effects of date, nest age and experimental group. Difference in hatching failure rate between experimental groups was analysed using a Chi-squared test. We defined hatching failure rate as the proportion of eggs surviving the incubation period that did not hatch^26^. Incubation period and hatching asynchrony were compared between experimental groups using *t*-tests.

We modelled hatchling body condition by fitting linear mixed models with biometric measurements (body mass, tarsus, bill, head and bill) of the chicks as response variables, experimental group, mean clutch-specific egg volume (to correct for effects of egg size) and laying date as fixed effects and brood ID as random effect. For chick growth data we compared three types of (sigmoid) growth curves for each biometric measurement (body mass, tarsus, bill, head and bill) across all broods using the Gompertz, logistic and Weibull growth functions. For all measurements the logistic growth curve was the best fit. We used residuals from the growth curve for each measurement as response variable in linear mixed models with experimental group, chick age, habitat, and laying date as fixed effects, and brood ID and chick ID as random effects. We included interaction terms between chick age and experimental group to test whether chicks from the enlarged group had a slower growth rate than controls, and between chick age and habitat to account for possible effects of (feeding) habitat on chick growth rate. We excluded data from chicks > 15 days old for this analysis, as we lacked data from experimentally enlarged clutches after this stage. This was due to the low number of chicks from the enlarged group surviving to this age (or reaching this age before the field season ended), combined with the increasing difficulty of locating and capturing chicks towards the later stages of the chick rearing period.

We used Cox proportional-hazards modelling from the R package “survival”^44^ to analyse chick survival. Brooding activity in Ringed Plovers markedly levels off when chicks reach 10–11 days of age^32^, making it harder to locate and recapture complete broods after this point. We therefore analysed hazard ratios of chicks up to an age of 10 days. We constructed a survival model with survival (0 = alive, 1 = dead/censored) as response variable, and experimental group, habitat and laying date as predictors. Brood ID was included as a random effect using the “frailty” function. Preliminary analyses indicated no significant effect of habitat or laying date, with the best supported model only including experimental group. As we lacked information about habitat for some of the chicks, these variables were removed to maximize sample size in the final analyses including experimental group and laying date.

We compared statistical models using the Akaike Information Criterion for small sample sizes (AICc). We checked competitive models (within 2 ΔAIC of the top model with the lowest AIC score)^45^ for uninformative parameters^46^ and chose the simplest alternative as our best supported model, but without removing parameters of importance to test our predictions. In AIC model selection, variables with *p* ≤ 0.157 will improve the AIC compared to a model with one fewer term and will be retained in the AIC-top model. To ensure consistency between AIC model selection and parameter evaluation criteria, we considered variables with 85% confidence intervals overlapping zero as uninformative^46,47^, and report 85% intervals along with the conventional 95% intervals.

To evaluate the combined effect of different measures of reproductive output throughout the breeding period, we calculated cumulative reproductive values (*R*)^10^ of clutches from experimental groups:

$$R = C{\text{ }}x{\text{ }}N{\text{ }}x{\text{ }}P{\text{ }}x{\text{ }}H$$

where *C* is clutch size, *N* is nest success, *P* is partial clutch survival and *H* is hatchability. We added the survival rate of chicks until age = 10 (*S10*) days (i.e., minimum proportion of chicks surviving their first 10 days of life) to this formula to calculate cumulative reproductive value up to this point (*R10*). *S10* could only be calculated for a reduced sample size as the families (adults and/or chicks) were not recovered or checked upon at this age, or because the field season ended before the chicks reached this age. For enlarged clutches, we used *C* = 5, treating the artificial egg as if it had been real and subject to the same survival probabilities as the rest of the clutch.

## Results

### Incubation and hatching

Of all nests included in the study, 6 control and 7 enlarged clutches were predated. There was no effect of experimental group or nest age on daily nest survival rate (Supplementary Table S1). We observed only one case of partial clutch loss, as one enlarged clutch lost two eggs.

We found a significant interaction between experimental groups and days from the onset of incubation on egg mass (linear term; Table 1; Supplementary Fig. S1), where egg mass declined at a lower rate in enlarged clutches than in control clutches. Incubation periods of enlarged clutches were significantly longer than for controls (control: 23.3 ± 1.34 days, *n* = 16; enlarged: 26.3 ± 1.65 days, *n* = 17; *t* = − 6.30, df = 28, *p* < 0.001; Supplementary Fig. S2A). The degree of hatching asynchrony was also larger in the enlarged clutches (control: 0.69 ± 0.25 days, *n* = 16; enlarged: 1.98 ± 1.22 days, *n* = 17; *t* = − 4.68, df = 24, *p* < 0.001; Supplementary Fig. S2B), but there was no statistically significant difference in hatching failure rates of eggs between enlarged and control clutches (control: 3.1 ± 8.5%, *n* = 64; enlarged: 10.3 ± 15.5%, *n* = 66; χ^2^ = 1.78, *p* = 0.18). However, the power to detect differences was limited (~ 25% at α = 0.05). Large embryos were present in 1 of 2 fully incubated but unhatched eggs in the control group, and 4 of 5 unhatched eggs from the enlarged group. The last two eggs contained no visible embryos.

**Table 1:** Parameter estimates (± SE) and confidence intervals (CI; 95% and 85%) from the best supported mixed effect model explaining egg mass loss with experimental group (control/enlarged; *n* = 18/18 clutches) and days since the onset of incubation (“age”), and the interaction between experimental group and days from the onset of incubation.

Variable			95% CI		85% CI		
	Estimate	SE	lower	upper	lower	upper	*p*
(Intercept)	1.036	0.006	1.025	1.048	1.028	1.045	< 0.001
Group	0.010	0.008	− 0.006	0.025	− 0.002	0.021	0.217
Age	− 0.007	1.83e–04	− 0.007	− 0.006	− 0.007	− 0.006	< 0.001
Age^2^	− 9.6e–05	3.9e−05	− 1.74e–04	− 1.90e–05	− 1.53e–04	− 3.96e–05	0.015
Group : Age	9.22e–04	2.66e–04	3.98e–04	0.001	5.38e–04	0.001	< 0.001
Group : Age^2^	1.60e–05	5.33e–05	− 8.89e–05	1.21e–04	− 6.09e–05	9.32e–05	0.764

### Hatchlings

**Fig. 1:**
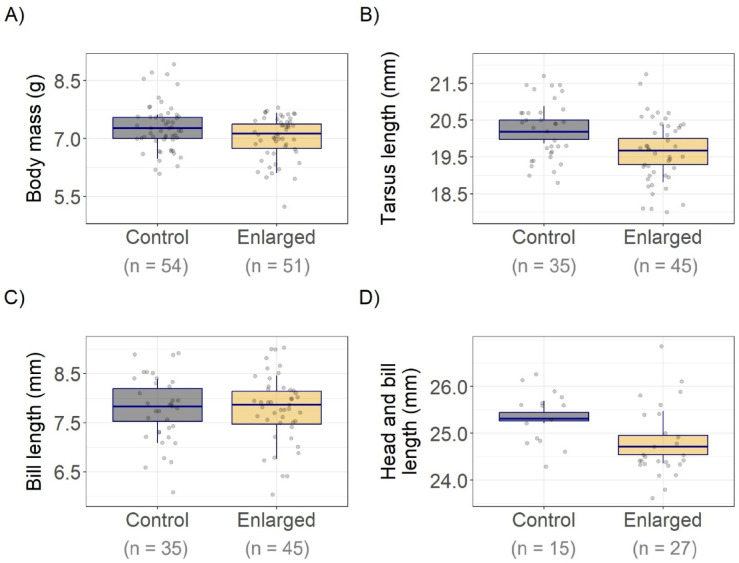
Effect of experimental group on (**A**) body mass, (**B**) tarsus length, (**C**) bill length and (**D**) total head (head and bill) length of Common Ringed Plover *Charadrius hiaticula* hatchlings (age ≤ 2 d) from control (4-egg) and experimentally enlarged (5-egg) clutches, with model predictions (box plots) and raw data (points). Box plots show medians (horizontal line within boxes) and interquartile range (lower and upper hinges) for hatchlings from control (4-egg) and experimentally enlarged (5-egg) clutches. Effect sizes are from the linear mixed models shown in Table 1.

Hatchlings from the enlarged group were lighter and had shorter head and bill length than controls (Fig. 1; Table 2). For tarsus length, the best supported model suggested a marginal effect of experimental group, with shorter tarsus length in hatchlings from 5-egg clutches (Table 2). No effect of experimental group was seen for bill length (Table 2). There were significant positive effects of egg volume in the best supported models for hatchling body mass and tarsus length (Supplementary Table S2), showing that heavier and larger chicks hatched from larger eggs (Table 2). Laying date had a significant positive effect on tarsus length and bill length (Table 2).

**Table 2:** Parameter estimates (± SE) and confidence intervals (CI; 95% and 85%) from the best supported linear mixed effect models explaining hatchling measurements (body mass (control/enlarged, *n* = 54/51 chicks), Tarsus (*n* = 35/45 chicks), bill (*n* = 35/45 chicks) and total head (*n* = 15/27 chicks) length) with candidate models including experimental group, egg volume and laying date as fixed factors and brood ID as random effects.

Response				95% CI		85% CI		
	Model	Estimate	SE	lower	upper	lower	upper	*p*
Mass	(Intercept)	1.363	1.075	− 0.796	3.565	− 0.202	2.952	0.215
	Group (enlarged)	− 0.301	0.131	− 0.569	− 0.037	− 0.494	− 0.110	0.029
	Egg volume	0.621	0.112	0.391	0.847	0.455	0.785	< 0.001
Tarsus	Intercept	15.04	1.682	11.56	18.44	12.54	17.50	< 0.001
	Group (enlarged)	− 0.367	0.204	− 0.780	0.053	− 0.666	− 0.065	0.085
	Egg volume	0.400	0.173	0.051	0.757	0.148	0.657	0.029
	Laying date	0.096	0.029	0.037	0.155	0.053	0.138	0.003
Bill	Intercept	6.401	0.461	5.450	7.330	5.717	7.074	< 0.001
	Group (enlarged)	0.242	0.208	− 0.182	0.667	− 0.064	0.548	0.257
	Laying date	0.090	0.029	0.032	0.150	0.048	0.133	0.004
Head and bill	Intercept	25.42	0.234	24.94	25.94	25.08	25.79	< 0.001
	Group (enlarged)	− 0.676	0.288	− 1.321	− 0.088	− 1.129	− 0.255	0.037

### Chick growth and survival

**Fig. 2:**
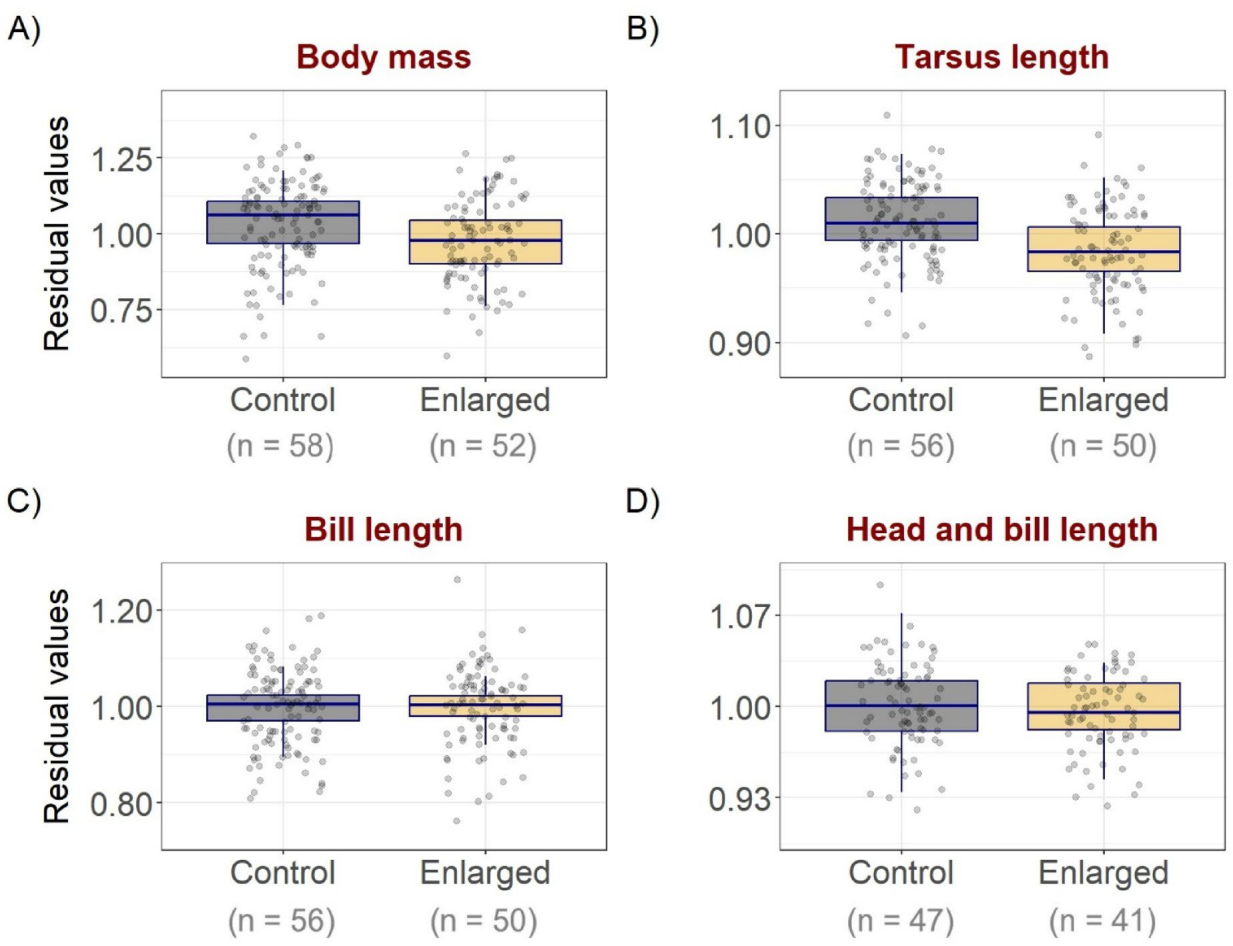
Effect of experimental group on (**A**) body mass, (**B**) tarsus length, (**C**) bill length and (**D**) total head (head and bill) length of Common Ringed Plover *Charadrius hiaticula* chicks during the chick-rearing period (age ≤ 15 d), with model predictions (box plots) and raw data residuals (points) from a logistic growth curve fitted to our data. Box plots show medians (horizontal line within boxes) and interquartile range (lower and upper hinges) for chicks from control (4-egg) and experimentally enlarged (5-egg) clutches. Effect sizes are from the linear mixed models shown in Table 2.

Chicks from enlarged clutches were consistently lighter, and had shorter tarsus length, than chicks from control clutches throughout the first 15 days post-hatching (Fig. 2; Table 3). No significant effects of experimental group was found on bill length or head and bill length (Fig. 2; Table 3). Each of the best supported models for body mass, tarsus length and bill length included a significant interaction between habitat and chick age (Supplementary Table S3), with chicks found in marine habitats growing faster than those staying in terrestrial habitats (Table 3). Laying date had a significant positive effect on bill length in the best supported model. (Table 3). We found no significant interaction between experimental group and chick age for any of the morphometrics, suggesting similar rates of growth in chicks from both groups (Supplementary Fig. S3; Supplementary Table S4).

**Table 3:** Parameter estimates (± SE) and confidence intervals (CI; 95% and 85%) from the best supported linear mixed effect models explaining chick growth measurements (body mass (control/enlarged, *n* = 58/52 chicks), Tarsus (*n* = 56/50 chicks), bill (*n* = 56/50 chicks) and total head (*n* = 47/41 chicks) length) with candidate models including fixed effects of experimental group, chick age, habitat and laying date, and interactions between habitat and chick age, and between experimental group and chick age, and random effects of brood ID and chick ID.

Response				95% CI		85% CI		
	Model	Estimate	SE	lower	upper	lower	upper	*p*
Mass	(Intercept)	1.130	0.030	1.070	1.191	1.086	1.175	< 0.001
	Group (enlarged)	− 0.084	0.039	− 0.163	− 0.006	− 0.141	− 0.027	0.038
	Habitat (marine)	− 0.221	0.033	− 0.287	− 0.154	− 0.270	− 0.172	< 0.001
	Age	− 0.013	0.002	− 0.018	− 0.008	− 0.017	− 0.010	< 0.001
	Habitat (marine) * Age	0.025	0.004	0.017	0.033	0.019	0.031	< 0.001
Tarsus	(Intercept)	1.028	0.008	1.012	1.045	1.016	1.041	< 0.001
	Group (enlarged)	− 0.036	0.011	− 0.058	− 0.015	− 0.052	− 0.021	0.002
	Habitat (marine)	− 0.030	0.009	− 0.049	− 0.011	− 0.044	− 0.016	0.002
	Age	− 0.003	6.9e–04	− 0.004	− 0.002	− 0.004	− 0.002	< 0.001
	Habitat (marine) * Age	0.005	0.001	0.003	0.008	0.004	0.007	< 0.001
Bill	(Intercept)	0.960	0.034	0.892	1.029	0.910	1.010	< 0.001
	Group (enlarged)	0.002	0.019	− 0.036	0.040	− 0.025	0.030	0.900
	Habitat (marine)	− 0.068	0.024	− 0.117	− 0.020	− 0.104	− 0.032	0.005
	Age	− 0.003	0.002	− 0.007	3.5e–04	− 0.006	− 0.001	0.075
	Laying date	0.005	0.002	3.6e–04	0.009	0.002	0.008	0.036
	Habitat (marine) * Age	0.007	0.003	3.8e–04	0.013	0.002	0.011	0.036
Head and bill	(Intercept)	1.003	0.006	0.100	1.016	0.994	1.013	< 0.001
	Group (enlarged)	− 0.009	0.009	− 0.028	0.010	− 0.023	0.004	0.328

**Fig. 3:**
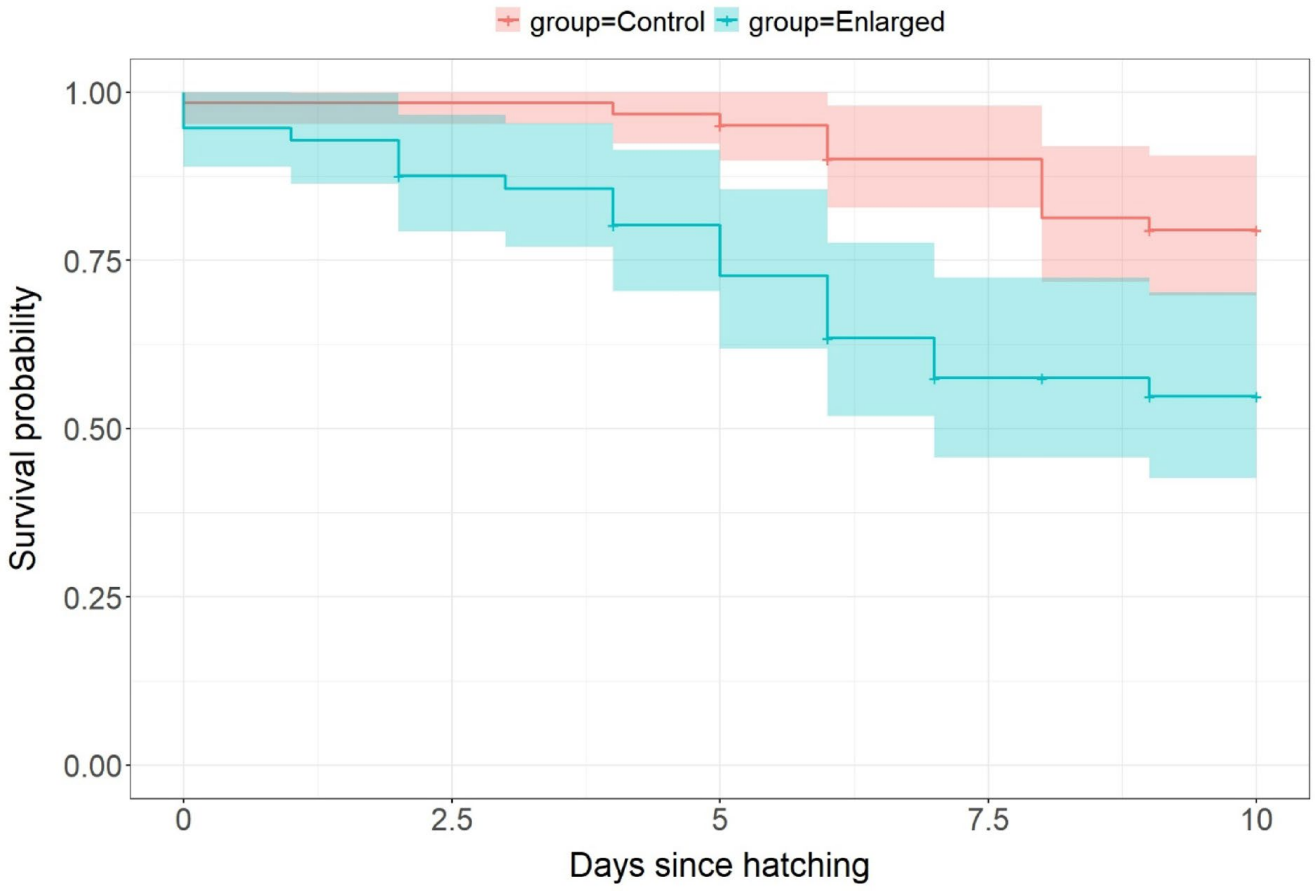
Survival curves with confidence intervals for chicks hatching from control (4-egg; *n* = 61 chicks) and enlarged (5-egg; *n* = 56 chicks) clutches in the Common Ringed Plover *Charadrius hiaticula* during their first 10 days of life. The survival curves do not take into account the (insignificant) random effect of brood ID included in the Cox Proportional Hazard model (see text).

Chicks from the enlarged group had a significantly (3.5 times, 95% CI [1.50–8.30]; *p* = 0.004) higher risk of death before reaching 10 days of age compared to chicks from the control group, with most hazards recorded during the first week after hatching (Fig. 3). The minimum proportion of chicks surviving their first 10 days of life was 78.9% in the control group (*n* = 57) and 51.3% in the enlarged group (*n* = 39).

### Cumulative experimental effects

**Fig. 4:**
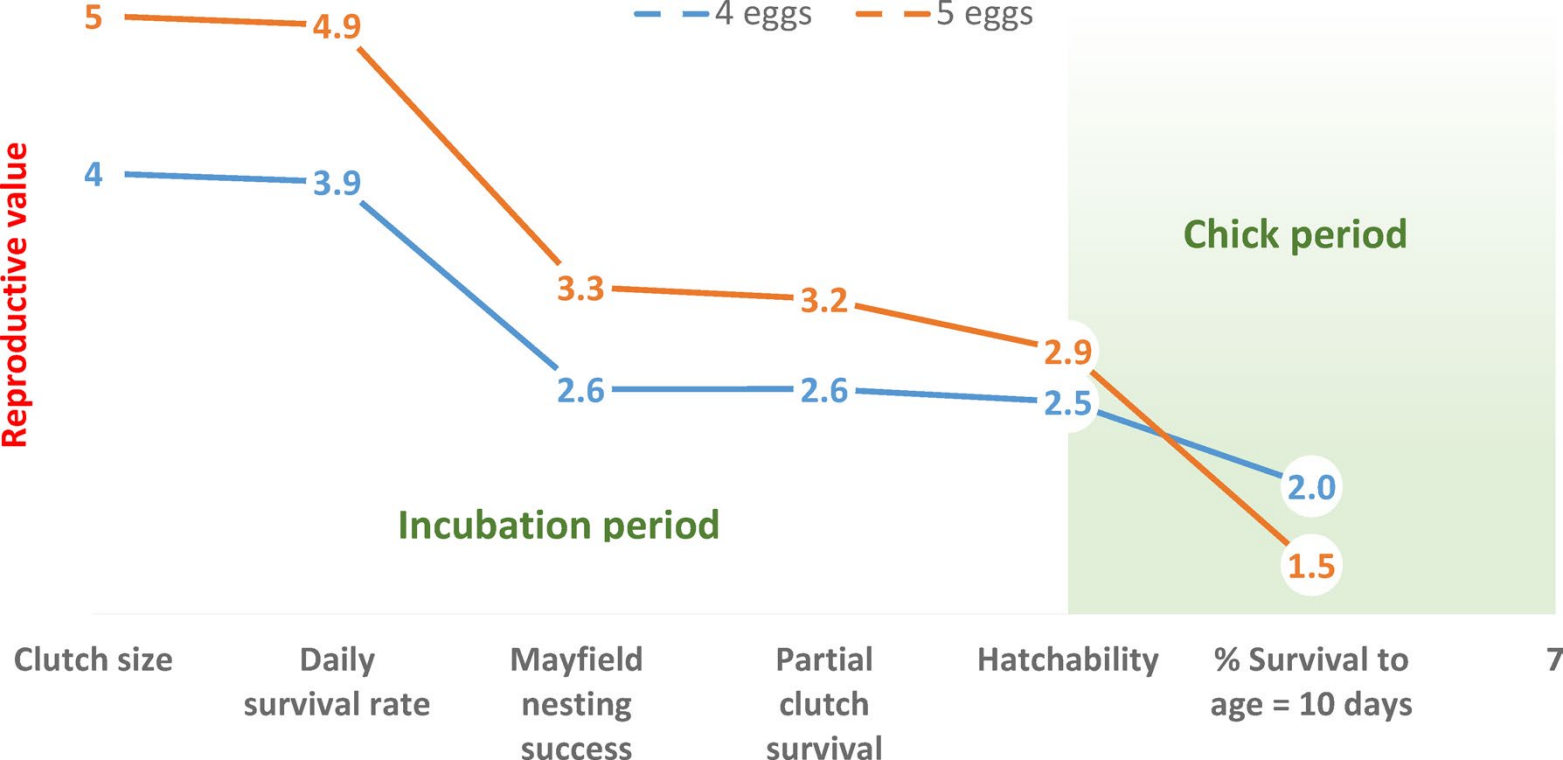
Cumulative reproductive value^10^ of control (4-egg) and enlarged (5-egg) clutches in the Common Ringed Plover *Charadrius hiaticula* when adding different measures of reproductive output throughout the breeding period. Each value is calculated as the product of the corresponding variable and the variable(s) to the left. For background values, see Table 3.

When considering the cumulative effects of clutch size, daily survival rates, Mayfield nesting success, partial clutch survival and hatching failure rates, the reproductive value of enlarged clutches was 11% higher than that of control clutches at the time of hatching, due to their larger initial clutch size and only small and insignificant differences in hatching success (Table 4). However, because chick mortality was much higher, the cumulative reproductive value of enlarged clutches dropped to 36% below that of controls 10 days after hatching (Fig. 4; Table 4).

**Table 4:** Effects of clutch size (C) on duration of nesting period (laying (Lay), incubation (Inc), and total; in days), daily nest survival rate (DSR), mayfield nesting success (N), partial clutch survival (P), hatchability (H), (minimum) chick survival (until age = 10 days; S10), and cumulative reproductive value (R = C x N x P x H; R2 = C x N x P x H x S10)^10^. Difference (Diff) refers to percent increase or decrease in reproductive value of enlarged clutches, with and without chick survival included. Setup of table is modified from Arnold^10^.

C	Duration				*N*^2^	*P*	H	S10	*R*	R2	Diff (*R*)	Diff (R2)
	Lay^1^	Inc^1^	Total	DSR								
4	6.5	23.3	29.8	0.986	0.657	1.000	0.969	0.789	2.546	2.010		
5	8.7	26.3	35.0	0.988	0.655	0.977	0.897	0.513	2.872	1.473	11%	− 36%

^1^: Estimates from Wallander and Andersson^28^, ^2^: N = DSR^n^, where n = total duration of the nesting period.

## Discussion

As predicted if clutch size is limited by parental incubation capacity in precocial birds, we here show that enlarging clutch size with a single egg may have profound negative effects on size, body condition, growth, and survival of shorebird chicks. We also found that these effects alone are sufficient to outweigh the reproductive surplus resulting from an extra egg, thus making supernormal clutches maladaptive even without the reduced reproductive value at hatching which was demonstrated by Arnold^10^. We did not measure egg temperatures, but our findings are largely consistent with effects which are expected if incubation temperatures are reduced in supernormal clutches^13,18,19,21^. Our study is the first to demonstrate such a direct link between increased clutch size and offspring growth and survival in a precocial bird.

A key result indicating limited incubation capacity in Ringed Plovers is that clutches experimentally enlarged with a single egg experienced prolonged incubation periods compared to normal control clutches. Similar findings have been previously reported from several species^10,11,48^, and likely result from lower incubation temperatures in larger clutches^11,49,50^. This relationship is typically attributed to reduced metabolism and slower development of the embryo^18,23^. Intra-clutch variation in incubation temperatures may also increase in larger clutches^11,49^, potentially explaining the asynchronous hatching observed in enlarged clutches in the present study. Such reduced or more variable incubation temperatures could result from at least two mechanisms: (1) lower incubation efficiency or (2) altered incubation behaviour. In a parallel study using video recordings of Ringed Plovers incubating four and five eggs we found preliminary support for the former mechanism, as the birds showed similar nest attendance and number of recesses between clutch sizes (unpubl. data).

Hatchlings from enlarged clutches showed both lower body mass and smaller structural size than controls. We expected this result if clutch enlargement led to lower incubation temperatures – an effect directly demonstrated in other studies^11,49,50^. Smaller body size at hatching can have crucial consequences for the chicks, as both body size and condition are critical for the survival and lifetime fitness of young shorebirds^51–53^. As expected (and in line with the findings of Székely, et al.^30^), we also observed a lower rate of weight loss in eggs from enlarged clutches than eggs from control clutches. This may indicate reduced embryonic metabolism and growth rate in larger clutches, likely caused by lower incubation temperatures as demonstrated in experimental studies^29^.

Despite the reduced weight loss rate, the longer incubation period in enlarged clutches likely resulted in continued weight loss over a longer time, which could have contributed to the smaller size of hatchlings in this group. Additionally, lower incubation temperatures may increase embryonic energy expenditure during hatching^29,54^. Combined with the longer incubation periods, this may have caused higher total energy use in embryos from enlarged clutches. Previous studies have shown negative relationships between incubation temperature and the proportion of yolk converted to hatchling tissue, resulting in lighter embryos and reduced residual yolk in hatchlings^20,29^. Thus, differences in residual yolk may also explain why hatchlings from five-egg clutches were smaller and lighter than those from four-egg clutches in our experiment. Since yolk serves as a vital energy and water reserve for neonates, increased metabolism during embryonic development could leave less yolk available post-hatching, potentially impairing early growth and survival^55,56^.

Only two previous studies have investigated the direct effects of clutch enlargement on hatchlings in shorebirds. As in Ringed Plovers, hatchlings of Northern Lapwings from clutches with five eggs had lower body mass than those from four-egg clutches^26^, but no effects on hatchling body mass and condition existed in Pied Avocets^27^. These differences between species could be related to the colonial breeding biology of Avocets with relatively high natural frequencies of supernormal clutches^57^. Thus, Avocets may be better able to efficiently incubate larger clutches than non-colonial shorebirds^27^. Furthermore, a limited incubation capacity may have more severe consequences for the embryos in cold (and very warm) climates, where poorly covered eggs should be more often exposed to suboptimal temperatures^6,58^. We therefore encourage more studies testing for effects of clutch size on fitness of shorebird chicks across climates and breeding strategies.

It is possible that young animals could compensate for being small early in life by growing faster at a later stage^59^, but no sign of such compensatory growth was found in the Ringed Plover chicks. Instead, chicks from experimentally enlarged clutches exhibited similar growth rates compared to controls during their first 15 days of life. Since their body condition and size were smaller at hatching, the chicks also remained smaller throughout our study period. In egg cooling experiments, low incubation temperatures have been found to increase the intensity of energy expenditure^23^ and reduce thermoregulatory performance in chicks^22,60^. Combined with a generally smaller size and thus higher surface-to-volume ratio this may have made chicks from enlarged clutches more in need of frequent brooding^61^, thereby reducing the time available for feeding^62^. Furthermore, the chicks may have been negatively affected by size- or energy-related reductions in feeding efficiency^22^. In either case, this would most likely reduce their potential for compensatory growth and lower their chances of survival.

Importantly, Ringed Plover chicks from enlarged clutches had a higher mortality rate than control chicks. From our discussion above, it seems possible that a consistently smaller body size in Ringed Plover chicks from enlarged clutches might have given them a poor start in life which they were not able to compensate for, and which led to the increased mortality risk. Chick survival may also have been reduced because lower incubation temperatures could suppress immunocompetence in chicks^51,63^, make them more vulnerable to stress^24,64^, alter their energy metabolism^23,24^ or lower their thermoregulatory ability^22,60^.

Our findings suggest that the potential benefit of laying an extra egg in a shorebird clutch may be outweighed by increased chick mortality. Consequently, because egg production is costly to females^2^, investing in an extra egg would often be a waste of resources, ultimately rendering supernormal clutches maladaptive. However, several other factors that we have not investigated here could also potentially contribute to, and thus reinforcing, clutch size limitation in shorebirds. First, there may be costs of egg production in females which could limit the optimal clutch size, for example if their energy budget are critically reduced by laying an extra egg^2,48^. Moreover, if nutrients are limited, this could result in reduced egg sizes and lower egg quality^65^, and thus reduced embryonic viability^58,65^. Second, it is possible that the additional costs of an extra egg in shorebirds are present at the brood stage, if for example parents are less able to adequately warm a brood of five chicks due to a limited brood patch size, and/or if a larger brood is more conspicuous to predators^66^. Furthermore, it is possible that an additional chick might increase intra-brood competition for food and other resources among siblings^27^. Consequently, survival rates should be further reduced, and the true costs of producing a five-egg clutch could be even greater than suggested by our estimates.

Finally, and since we did not cross-foster chicks between experimental groups at the time of hatching, indirect negative effects on the incubating parents cannot be ruled out in our study. Incubating an enlarged clutch may require greater parental efforts involving increasing energetic expenditure or stress during incubation^48^. This could reduce the parents’ ability to provide optimal care and/or occupy the best feeding areas for their chicks^27,48,51^, thereby contributing to higher chick mortality. Nevertheless, the prolonged incubation periods, reduced rate of egg weight loss, more asynchronous hatching, and the smaller-sized hatchlings seen in the enlarged group strongly suggest that limitations during incubation played a major role in our study. Nonetheless, we cannot rule out the possibility that both mechanisms may have been involved and contributed to the high mortality of chicks hatching from enlarged clutches.

To conclude, our results are consistent with the hypothesis proposed by Lack^1^ that clutch sizes are constrained by the limited incubation capacity of parents in precocial birds with a relatively fixed maximal number of eggs laid per clutch, such as the shorebirds. The lower structural size and body condition of Ringed Plover chicks from enlarged clutches, and especially the higher mortality rates of these chicks in early life, caused a lower overall reproductive value than in control clutches. However, we acknowledge that costs associated with life-history trade-offs could contribute to the observed patterns, and these mechanisms cannot be fully separated without additional experimental approaches such as cross-fostering. In addition, more studies are needed to investigate how general our findings are across taxa. These studies should test the effects of clutch enlargement on chicks in species with different breeding systems and varying levels of parental care, and which live in diverse climates and environments. We also encourage more studies specifically testing for effects of clutch enlargement on egg temperature in shorebirds, and mechanisms through which these could carry over to affect phenotypes and survival probability in chicks.

## Supplementary Information

Below is the link to the electronic supplementary material.

Supplementary Material 1

## Data Availability

The data and code generated during the current study have been archived in Figshare, 10.6084/m9.figshare.29715869.
