# Supplementary material for: Reduced chick performance makes supernormal clutches maladaptive in a shorebird
Source: Sci Rep. 2026 Feb 4;16:7305. doi: 10.1038/s41598-026-37872-6 (PMC12923721; doi:10.1038/s41598-026-37872-6)
Supplement: Supplementary file 1 — Supplementary Material 1 [file 41598_2026_37872_MOESM1_ESM.docx]

## Supplementary Information

**Reduced chick performance makes supernormal clutches maladaptive in a shorebird**

Oddvar Heggøy*^1^, Kees Wanders^2,3^, Terje Lislevand^1^

*: corresponding author; [oddvar.heggoy@uib.no](mailto:oddvar.heggoy@uib.no)

^1^: Department of Natural History, University Museum of Bergen, University of Bergen, Bergen, Norway; ^2^: Natural History Museum of Denmark, University of Copenhagen, Copenhagen, Denmark; ^3^: Department of Life Sciences, Milner Centre for Evolution, University of Bath, Bath, UK

**Table S1.** Modelling results for daily survival rates of nests of Common Ringed Plover *Charadrius hiaticula* with nest age, date and experimental group as predictors.

| Model | AICc | **ΔAICc** | weight | *n* parameters | Deviance |
| --- | --- | --- | --- | --- | --- |
| - | 99.59 | 0.00 | 0.40 | 1 | 97.58 |
| Nest age | 101.10 | 1.51 | 0.19 | 2 | 97.09 |
| Date | 101.11 | 1.52 | 0.19 | 2 | 97.09 |
| Group | 101.57 | 1.98 | 0.15 | 2 | 97.56 |

**Table S2.** Model selection results for morphological measurements of Common Ringed Plover *Charadrius hiaticula* hatchlings from control (4-egg) and enlarged (5-egg) clutches, using linear mixed-effect models including a random effect for brood to control for non-independence of repeated measurements. Only models with an effect for experimental group were considered. Models are ordered from smallest to highest ΔAIC, and only models with ΔAIC < 3 are shown.

| **Response** | **Model** | **AICc** | **ΔAICc** | **weight** |
| --- | --- | --- | --- | --- |
| body mass | **group + egg volume** | 129.58 | 0.00 | 0.61 |
|  | group + egg volume + laying date | 130.45 | 0.88 | 0.39 |
| tarsus length | **group + egg volume + laying date** | 192.06 | 0.00 | 0.76 |
|  | group + laying date | 194.65 | 2.59 | 0.21 |
| bill length | **group + laying date** | 152.36 | 0.00 | 0.68 |
|  | group + egg volume + laying date | 154.19 | 1.82 | 0.27 |
| head and bill length | **group** | 93.18 | 0.00 | 0.51 |
|  | group + laying date | 95.06 | 1.88 | 0.20 |
|  | group + egg volume | 95.09 | 1.91 | 0.20 |

**Table S3.** Model selection results for chick growth measurements of Common Ringed Plover *Charadrius hiaticula* from control (4-egg) and enlarged (5-egg) clutches, using linear mixed-effect models with candidate models including fixed effects of experimental group, chick age, habitat and laying date, and interactions between habitat and chick age, and between experimental group and chick age, and random effects of brood ID and chick ID. Only models with an effect for experimental group were considered. Models are ordered from smallest to highest ΔAIC, and only models with ΔAIC < 3 are shown.

| **Response** | **Model** | **AICc** | **ΔAICc** | **weight** |
| --- | --- | --- | --- | --- |
| body mass | group + age + habitat + laying date + age:habitat | -325.8 | 0.00 | 0.48 |
|  | **group + age + habitat + age:habitat** | -324.6 | 1.17 | 0.27 |
|  | group + age + habitat + laying date + age:group + age:habitat | -323.6 | 2.19 | 0.16 |
| tarsus length | **group + age + habitat + age:habitat** | -832.6 | 0.00 | 0.46 |
|  | group + age + habitat + age:group + age:habitat | -831.6 | 0.99 | 0.28 |
|  | group + age + habitat + laying date + age:habitat | -830.5 | 2.13 | 0.16 |
| bill length | **group + age + habitat + laying date + age:habitat** | -472.4 | 0.00 | 0.35 |
|  | group + age + habitat + laying date + age:group + age:habitat | -470.4 | 1.95 | 0.13 |
|  | group + age + habitat + laying date | -470.2 | 2.16 | 0.12 |
|  | group + age + habitat + age:habitat | -470.1 | 2.29 | 0.11 |
| head and bill length | **group** | -722.9 | 0.00 | 0.16 |
|  | group + age + habitat + age:habitat | -722.6 | 0.27 | 0.14 |
|  | group + age | -722.4 | 0.43 | 0.13 |
|  | group + age + habitat | -721.8 | 1.07 | 0.10 |
|  | group + age + laying date | -721.2 | 1.63 | 0.07 |
|  | group + age + group:age | -721.2 | 1.69 | 0.07 |
|  | group + age + habitat + laying date + age:habitat | -721.1 | 1.72 | 0.07 |
|  | group + age + habitat + age:group + age:habitat | -721.0 | 1.87 | 0.06 |
|  | group + age + habitat + laying date | -720.6 | 2.23 | 0.05 |
|  | group + age + laying date + age:group | -720.1 | 2.74 | 0.04 |
|  | group + age + habitat + age:group | -720.1 | 2.75 | 0.04 |

**Table S4.** Parameter estimates (± SE) and confidence intervals (CI; 95% and 85%) from the best supported linear mixed effect models explaining chick growth measurements (body mass (control/enlarged, *n* = 58/52 chicks), tarsus (*n* = 56/50 chicks), bill (*n* = 56/50 chicks) and total head (*n* = 47/41 chicks) length) in Common Ringed Plover *Charadrius hiaticula.* Candidate models included fixed effects of experimental group, chick age, habitat and laying date, and interactions between habitat and chick age, and between experimental group and chick age, and random effects of brood ID and chick ID. Only models including the interaction between chick age and experimental group are shown.

|  |  |  | **95% CI** |  | **85% CI** |  |  |
| --- | --- | --- | --- | --- | --- | --- | --- |
| **Variable** | **Estimate** | **SE** | **lower** | **upper** | **lower** | **upper** | **p** |
| **Mass** |  |  |  |  |  |  |  |
| (Intercept) | 1.230 | 0.061 | 1.107 | 1.353 | 1.141 | 1.319 | < 0.001 |
| Group (enlarged) | -0.094 | 0.042 | -0.177 | -0.010 | -0.154 | -0.033 | 0.029 |
| Habitat (marine) | -0.217 | 0.033 | -0.283 | -0.149 | -0.265 | -0.167 | < 0.001 |
| Age | -0.013 | 0.003 | -0.019 | -0.008 | -0.017 | -0.009 | < 0.001 |
| Laying date | -0.008 | 0.004 | -0.016 | 5.9e-04 | -0.014 | -0.002 | 0.069 |
| Group (enlarged) : Age | 3.3e-04 | 0.004 | -0.007 | 0.008 | -0.005 | 0.006 | 0.930 |
| Habitat (marine) : Age | 0.025 | 0.004 | 0.016 | 0.033 | 0.019 | 0.031 | < 0.001 |
| **Tarsus** |  |  |  |  |  |  |  |
| Intercept | 1.025 | 0.009 | 1.008 | 1.043 | 1.012 | 1.038 | < 0.001 |
| Group (enlarged) | -0.030 | 0.012 | -0.054 | -0.005 | -0.048 | -0.012 | 0.018 |
| Habitat (marine) | -0.029 | 0.009 | -0.047 | -0.010 | -0.042 | -0.015 | 0.003 |
| Age | -0.003 | 7.8e-04 | -0.004 | -0.001 | -0.004 | -0.001 | 0.001 |
| Group (enlarged) : Age | -0.001 | 0.001 | -0.003 | 0.001 | -0.003 | 4.0e-04 | 0.273 |
| Habitat (marine) : Age | 0.006 | 0.001 | 0.003 | 0.008 | 0.004 | 0.007 | < 0.001 |
| **Bill** |  |  |  |  |  |  |  |
| Intercept | 0.956 | 0.035 | 0.887 | 1.026 | 0.905 | 1.007 | < 0.001 |
| Group (enlarged) | 0.010 | 0.025 | -0.039 | 0.059 | -0.026 | 0.046 | 0.684 |
| Habitat (marine) | -0.068 | 0.024 | -0.117 | -0.019 | -0.104 | -0.032 | 0.006 |
| Age | -0.003 | 0.002 | -0.007 | 0.001 | -0.006 | 2.3e-04 | 0.192 |
| Laying date | 0.005 | 0.002 | 3.4e-04 | 0.009 | 0.002 | 0.008 | 0.037 |
| Group (enlarged) : Age | -0.001 | 0.003 | -0.007 | 0.004 | -0.006 | 0.003 | 0.629 |
| Habitat (marine) : Age | 0.007 | 0.003 | 4.8e-04 | 0.013 | 0.002 | 0.011 | 0.034 |
| **Head and bill** |  |  |  |  |  |  |  |
| Intercept | 1.005 | 0.007 | 0.990 | 1.019 | 0.994 | 1.015 | < 0.001 |
| Group (enlarged) | -0.005 | 0.011 | -0.026 | 0.016 | -0.020 | 0.011 | 0.643 |
| Age | -1.9e-04 | 5.2e-04 | -0.001 | 8.3e-04 | -9.4e-04 | 5.6e-04 | 0.713 |
| Group (enlarged) : Age | 7.6e-04 | 7.9e-04 | -0.002 | 8.0e-04 | -0.002 | 3.8e-04 | 0.335 |


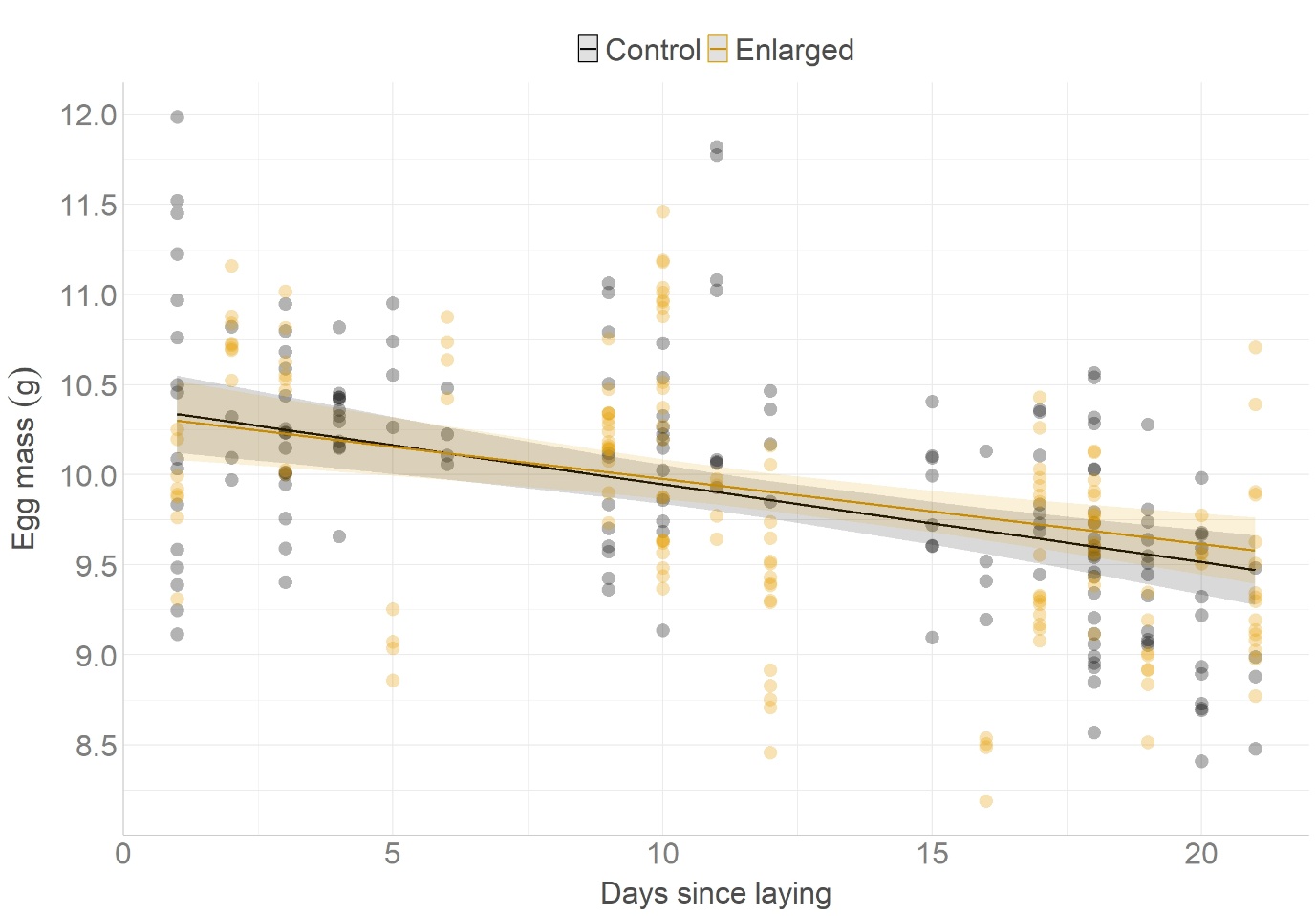


**Figure S1.** Mass loss rate of eggs from control (4-egg; *n* = 18) and enlarged (5-egg; *n* = 18) clutches in the Common Ringed Plover *Charadrius hiaticula*. Effect sizes are from the mixed model shown in Table S2.


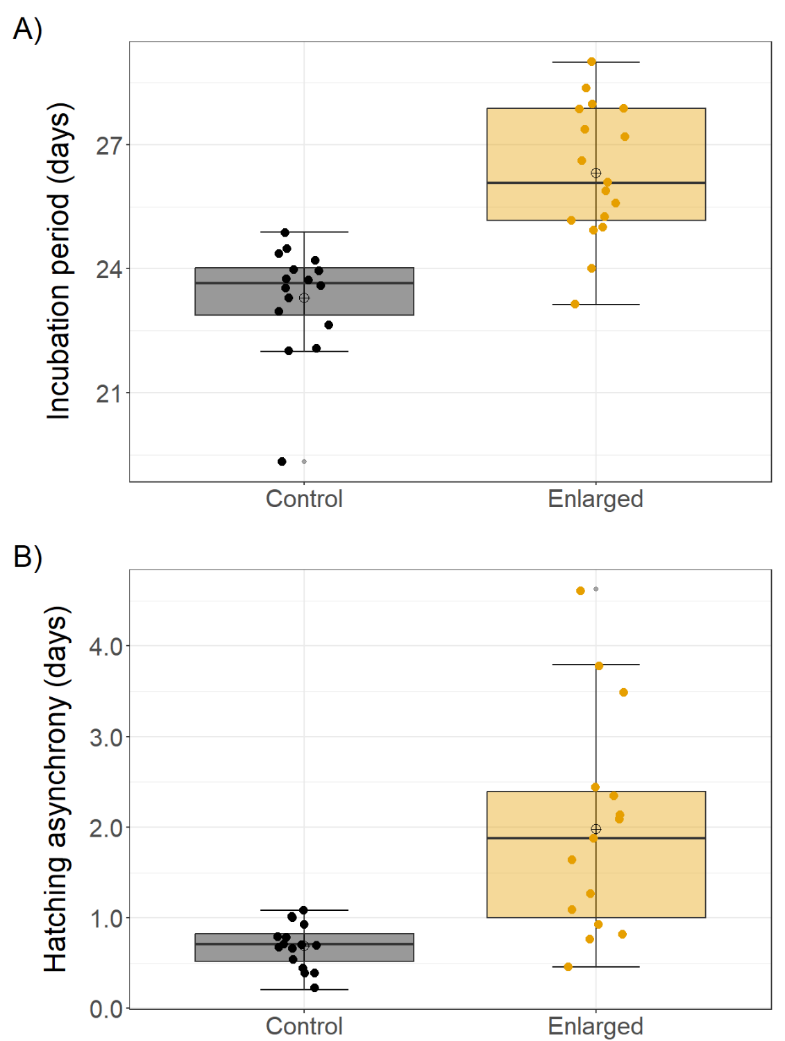


**Figure S2.** A) Incubation period and B) hatching asynchrony of control (4-egg; grey; *n* = 16) and experimentally enlarged (5-egg; yellow; *n* = 17) clutches in the Common Ringed Plover *Charadrius hiaticula*. Plots show median values (horizontal line within boxes) and interquartile range (lower and upper hinges) along with actual data points (dots).


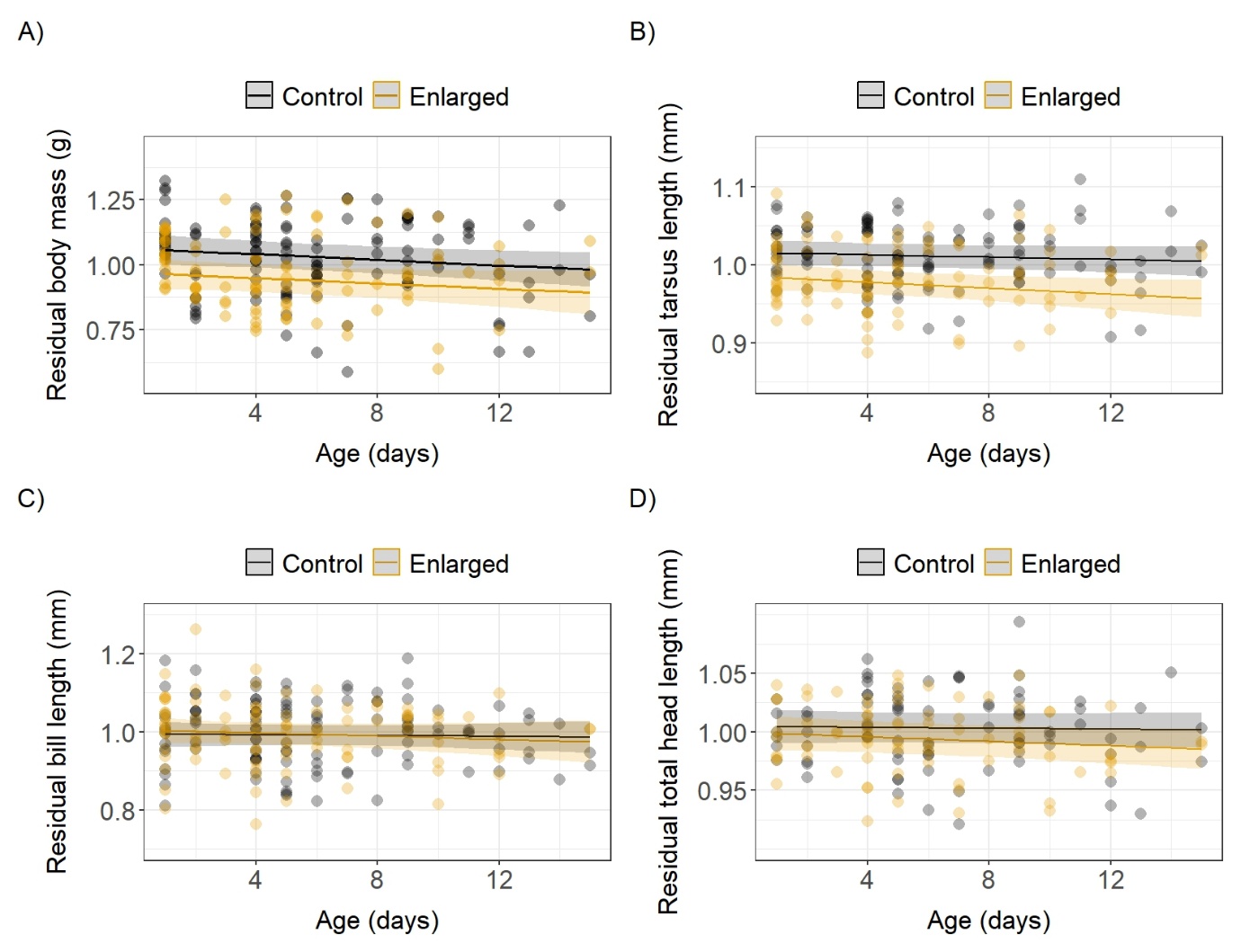


**Figure S3.** Growth of Common Ringed Plover *Charadrius hiaticula* chicks from control (4-egg) and experimentally enlarged (5-egg) clutches. Plots show observed values of A) body mass, B) tarsus length, C) bill length and D) total head (head and bill) length compared to values predicted by a logistic growth curve fitted to our data. Regression lines visualize the interaction between measurements and age (if any). Effect sizes are from the linear mixed models shown in Table A3.
